# The Evaluation of Cognitive Impairment in Alcohol-Dependent Patients Through RBANS Combined With ERPs

**DOI:** 10.3389/fpsyt.2020.598835

**Published:** 2021-01-21

**Authors:** Hui Cao, Chao Hou, Saiping Huang, Xiafeng Zhou, Jun Yang, Jia Bin Xu, Xiaoyun Cao, Zhenguang Li, Wei Zhang, Mei Zhang, Xuejun Liu, Xuhui Zhou

**Affiliations:** ^1^Department of Psychiatry, The Second People's Hospital of Hunan Province, The Hospital of Hunan University of Chinese Medicine, Changsha, China; ^2^Department of Psychiatry, Mental Hospital of Jiangxi Province, Nanchang, China; ^3^Department of Electrophysiology, The Second People's Hospital of Hunan Province, The Hospital of Hunan University of Chinese Medicine, Changsha, China; ^4^Department of Radiology, The Second People's Hospital of Hunan Province, The Hospital of Hunan University of Chinese Medicine, Changsha, China

**Keywords:** alcohol dependence patient, repeatable battery neuropsychological status (RBANS), event-related potentials, cognitive impairment, evaluation

## Abstract

**Background:** Recently, the cognitive impairment of patients with alcohol dependence has attracted more and more attention. The combination of Repeatable Battery for the Assessment of Neuropsychological Status (RBANS) and event-related potentials (ERPs) for evaluating the degree of cognitive impairment in patients with alcohol dependence has not undergone enough in-depth investigation.

**Method:** Sixty patients with alcohol dependence were selected as alcohol-dependence group, whereas 40 healthy volunteers served as a normal control group. The original scores of the RBANS sub-items, the incubation period, and volatility of ERPs between the two groups were compared, and the correlation among the above indicators in the alcohol-dependence group was further analyzed.

**Results:** The RBANS test showed that the original scores of speech function, attention function, delayed memory, and immediate attention in the alcohol-dependence group were significantly lower than those in the normal control group. Compared with the normal control group, the latencies of P200 and P300 in the alcohol-dependence group were significantly prolonged, and the amplitude of P200 and P300 was significantly reduced. Correlation analysis between RBANS and ERPs in alcohol-dependence group showed that immediate attention score was positively correlated with P300 and P200 amplitude, visual breadth score was positively correlated with P200 latency, and attention function score was negatively correlated with P300 latency.

**Conclusion:** As RBANS scale was highly correlated with the results of ERPs, the combined use of these two scales may serve as an objective basis for early diagnosis of cognitive impairment in patients with alcohol dependence.

## Introduction

Alcohol is a neurosuppressant that passes through the blood–brain barrier with a neurotoxic effect. Long-term alcohol use disorder (AUD) leads to lower blood flow to the frontal lobe, hippocampus, decreased oxygen metabolism, and reduced volume, which eventually leads to neuronal damage. This is the main cause of behavioral changes and cognitive impairment in clinically dependent patients (1, 2). Alcohol dependence has become one of the most serious public health problems. It was estimated that 50–80% of alcohol-dependent people had a certain degree of cognitive impairment (3), mainly in terms of memory, executive ability, visual spatial tasks, attention, learning ability, etc. (4). The cognitive impairment of alcohol-dependent people has serious adverse effects on the patient's social function and quality of life (5). Early detection of cognitive impairment in patients and early intervention can effectively delay the progression of disease in alcohol-dependent people. Therefore, the early assessment of cognitive impairment in alcohol-dependent people is an important part in controlling the harm caused by alcohol.

Currently, neuropsychological scales, including Repeatable Battery for the Assessment of Neuropsychological Status (RBANS), the Mini-Mental State Examination, Montreal Cognitive Assessment, and functional neuroimaging techniques (6–9), are used for the cognitive function assessment of alcohol-dependent people. Among them, RBANS, which is used to assess neuropsychological state, is sensitive to cognitive state in an equivalent form, taking a short time about 20 to 30 min (10). Originally used as a cognitive screening tool for people with Alzheimer disease, RBANS has been extended to a variety of neurological and mental disorders including AUD samples (11, 12). However, owing to operation of the test subjects, the educational levels, and compliance of the testee, the cognitive function of alcohol-dependent patients assessed by the neuropsychological test scale by far may have certain subjectivity and bias, so it is unable to accurately and effectively reflect the cognitive function of the subjects. Therefore, to carry out a comprehensive quantitative assessment, the use of objective examination for the cognitive function of alcohol-dependent people is the premise of early diagnosis.

With the development of cognitive neuroscience, scholars have applied various objective evaluation tools to reveal the characteristics of neurocognitive impairment in patients with alcohol dependence. Event-related potentials (ERPs) are highly sensitive in identifying neurocognitive impairments even when no behavioral impairment is detected (13). As a more objective and sensitive electrophysiological index, it exhibits the abnormality of brain electrical activity and then reflects the change of cognitive function. Therefore, ERPs have obvious advantages and irreplaceability clinically. The classic (narrow) ERPs are mainly composed of the exogenous components P100, N100, and P200 and the endogenous components N200 and P300 (14, 15). The main measurement indices in cognitive function studies are the latent period and amplitude, which represent the degree of effective resource participation in the brain's perception of input information. Among them, P300 is a representative index to record the cognitive processing process, which provides an important basis for clinical diagnosis and treatment (16). Usually induced by the classical oddball paradigm, P300 reflects complex psychocognitive activities. It is generally accepted that the decrease in the P300 amplitude is a sign of susceptibility to AUD (17). The P300 latent period is an evaluation of the brain's cognitive processing of information, which reflects brain efficiency to a certain extent. However, at clinical level, although ERPs is clearly highly sensitive and predictive, its specificity is poor (18).

In the past, many scholars discussed the cognitive impairment of alcohol dependence via neuropsychological tests and ERPs (19–22), and abnormalities in the ERPs could be a candidate of specific neuropsychological trait marker for AUD people. Although several of them have combined neuropsychological tests with ERP assessment as a comprehensive tool to assess alcohol-dependent cognitive function and to explore the correlation between clinical phenomenology and neuroelectrical physiology, they either did not consider the educational level of cases or did not recruit enough subjects. Specifically, apart from making up for the deficiencies in previous studies, this is the first study using two evaluation tools simultaneously to conduct two-angle testing of cognitive impairment of alcohol dependence in Chinese, aiming to link the clinical characteristics of cognitive impairment with characteristics of neuroelectrical physiology, thus revealing the neuropsychological and electrophysiological mechanisms of cognitive impairment of alcohol dependence and providing evidence-based support for early diagnosis and treatment of alcohol dependence.

In this study, the cognitive impairments of alcohol-dependent patients were evaluated by the combined scale of RBANS and ERPs. We also analyzed the correlation between the two evaluation indicators, which identified whether the RBANS scale and ERPs could be verified with each other and provided an objective basis for early identification and diagnosis of cognitive impairment of alcohol-dependent people.

## Methods

### Subjects

Alcohol-dependent patients in the inpatient department of the Psychiatry Department of the Second People's Hospital of Hunan Province from October 2017 to February 2018 were selected. Inclusion criteria were as follows: (1) all cases met the diagnostic criteria for alcohol dependence in the 4th Revision (DSM-IV-TR) of the *Diagnostic and Statistical Manual of Mental Disorders*; (2) those whose education level was junior high school or greater, aged 18–60 years, Han nationality; (3) those who completed acute withdrawal treatment for 7 days with no withdrawal symptoms. Exclusion criteria were as follows: (1) those having past and current history of brain injury, cerebral mental illness and other mental disorders; (2) those having substances other than nicotine dependence prior to entering the group; (3) those having consciousness disorders and delirium; (4) those who could not cooperate with the examination because of severe heart, liver, and kidney dysfunction; (5) pregnant and lactating women; and (6) those who exited the experiment.

The normal controls who met the inclusion criteria were the fixed or temporary employees of the Second People's Hospital of Hunan Province. Inclusion criteria were as follows: (1) those whose education level were junior high school or greater, aged 18–60 years, Han nationality; (2) all subjects had no history of alcohol dependence and a history of mental illness. Exclusion criteria were as follows: (1) those having past and current history of brain injury, cerebral mental illness, and other mental disorders; (2) those having substance other than nicotine dependence prior to entering the group; (3) those having consciousness disorders and delirium; (4) those who could not cooperate with the examination because of severe heart, liver, and kidney dysfunction; (5) pregnant and lactating women; and (6) those who withdrew from the experiment. This study informed both groups of subjects consenting to participate and was approved by the Ethics Committee of the Second People's Hospital of Hunan Province. Self-made general information of these recruited subjects included general demographic characteristics, average daily alcohol consumption, alcohol dependence years, alcohol dependence family history, and the daily amount of smoking.

Written informed consent was obtained from subjects recruited in both the alcohol-dependence group and the health control group, and the clinical trial passed the review of the Ethics Committee of the Second People's Hospital of Hunan Province.

### RBANS Scale

The study used RBANS (Randolph 1998 version) (23) with a total of 12 entries, which were digital breadth, coding test, picture naming, word fluency test, graphic reproduction, line positioning, vocabulary learning, story learning, vocabulary recall, vocabulary re-recognition, story recall, and graphical recall. RBANS can evaluate the cognitive level of the normal population and the degree of impairment of patients' cognitive function. The scale is widely used in the study of cognition abroad. This 30 min test is conducted in a quiet and undisturbed environment. Two alcohol addiction and Internet addiction physicians who have experienced the rating scale evaluation training will evaluate the subjects simultaneously. The correlation efficient between two physicians assessing the subjects is 0.93. Among them, a physician is randomly selected to inform the subjects of the instructions for each item. A clear and uniform speech of physician is required to ensure that subjects hear all of the instructions clearly. Two doctors are responsible for recording the original scores of 12 items simultaneously and finally take the mean value of the original scores of the 12 items. Twelve mean values were then transformed into five scale scores consisting of immediate memory, visual breadth, speech function, attention function, and delayed memory as the RBANS score for subjects. All subjects completed the test calmly, and the test will be canceled once abnormal performances, such as dizziness and vomiting, appear in subjects.

### ERPs Assays and Its Determination

Both the studies of alcohol-dependence group and the health control group were completed in the soundproof environment of the electrophysiological room. The room temperature was maintained at around 25°C. First, the experimental process was explained to the subjects, and the subjects were asked to take a seat and keep clear-headed and relaxed. Pretests including all the procedures in the formal experiment mentioned below were conducted before each formal examination, and the subjects began the formal experiments after they mastered the requirements of the experiment. In this study, the MEB9200K detector of Japanese optoelectronics was selected for the data recording, which placed the recording electrode on the Cz point. The reference electrode was double earlobe (A1, A2). The front of the head was grounded by the center patch electrode. All the electrode impedance was <5 KΩ. Stimulation was induced in the oddball paradigm. A pseudorandom sequence of deviant stimuli (15%) and standard stimuli (85%) was presented binaurally by a STIM 2 sound generator (Compumedics, El Paso, TX, USA), and 300 stimuli were presented binaurally through earphones. Each stimulus had a duration of 100 ms (10 ms rise and fall times) with uniform intertrial intervals of 1,250 ms. Target stimulation and non-target stimulation were 2,000 Hz, 80 dB of pure sound, and 1,000 Hz, 60 dB of pure sound, respectively. The probability ratio of target stimulation and non-target stimulation was 1/5. The subjects were asked to do the key press reaction responding to the target stimulus. The subjects with response time of <800 ms were averaged and analyzed, and those with response times of more than 800 ms were considered error responses. The analysis index was the incubation period and amplitude of P300 and P200, and the incubation period of N100 and N200 at Cz point. Component peaks were identified as the maximum voltage within the ranges as follows: N100 (maximum negative voltage from auditory tone to 150 ms), N200 (maximum negative voltage from 150 to 350 ms), and P300 (maximum positive voltage from 250 to 600 ms). Curry 7 software (Compumedics) was then used for processing electrophysiological signals offline. Recordings were down-sampled to 250 Hz, and data were then filtered using a referenced frequency of 0.3 to 30 Hz. Data were further segmented into 1,000-ms epochs, which all segments with voltage >±70 μV were automatically discarded.

### Statistical Analysis

All data were analyzed by the SPSS Statistics 23.0 statistical software and R project (version 3.6.1, https://www.r-project.org/). The measurement data were represented in $\bar{X}$ ± S. The independent-sample *t*-test was used to compare RBANS cognitive function score, ERP amplitude, and incubation period between the alcohol-dependent group and the normal group. The correlations between the indicators were analyzed by partial correlation analysis using R package ggm. The visualization of significantly correlated indicators was performed by R package ggplot2 and ggstatsplot. *P* < 0.05 was considered statistically significant. After adjusting by Bonferroni correction in the independent-sample *t*-test, *P*-values divided by the number of comparisons were considered statistically significant.

## Results

### The General Information of Patients

The alcohol-dependence group had 60 male cases, with an average age of 42.33 ± 7.57 years. The control group had 40 male cases, with an average age of 42.03 ± 6.61 years. The alcohol-dependence group had an average age at first drinking of 16.83 ± 2.14 years, an average daily drinking of 217.7 ± 32.63 g/d alcohol, average alcohol dependence years of 11.30 ± 6.94 years, and an average alcohol dependence questionnaire score of 20.13 ± 6.21. Compared with the general data of the alcohol-dependence group and the normal group, the differences between the age, the years of education, and the daily smoking volume group are not statistically significant (*P* > 0.05), as shown in Table 1.

**Table 1:** General demographic data and alcohol use in the alcohol-dependence group and the normal control group.

	**Alcohol-dependence group (*n* = 60)**	**Normal control group (*n* = 40)**	***F***	***p***
Age (year)	42.33 ± 7.57	42.03 ± 6.61	0.044	0.834
Education (year)	9.00 ± 1.85	9.58 ± 2.11	2.058	0.155
Age at first drinking (year)	16.83 ± 2.14	—	—	—
Daily drinking converted into pure alcohol (g/d)	217.7 ± 32.63	—	—	—
Alcohol dependence years (year)	11.30 ± 6.94	—	—	—
Alcohol dependence questionnaire score (#)	20.13 ± 6.21	—	—	—
The daily amount of smoking (stick/d)	22.28 ± 10.34	21.03 ± 9.68	0.355	0.553

### Comparison of RBANS Original Score Between the Alcohol Dependence Group and the Normal Control Group

**Figure 1:**
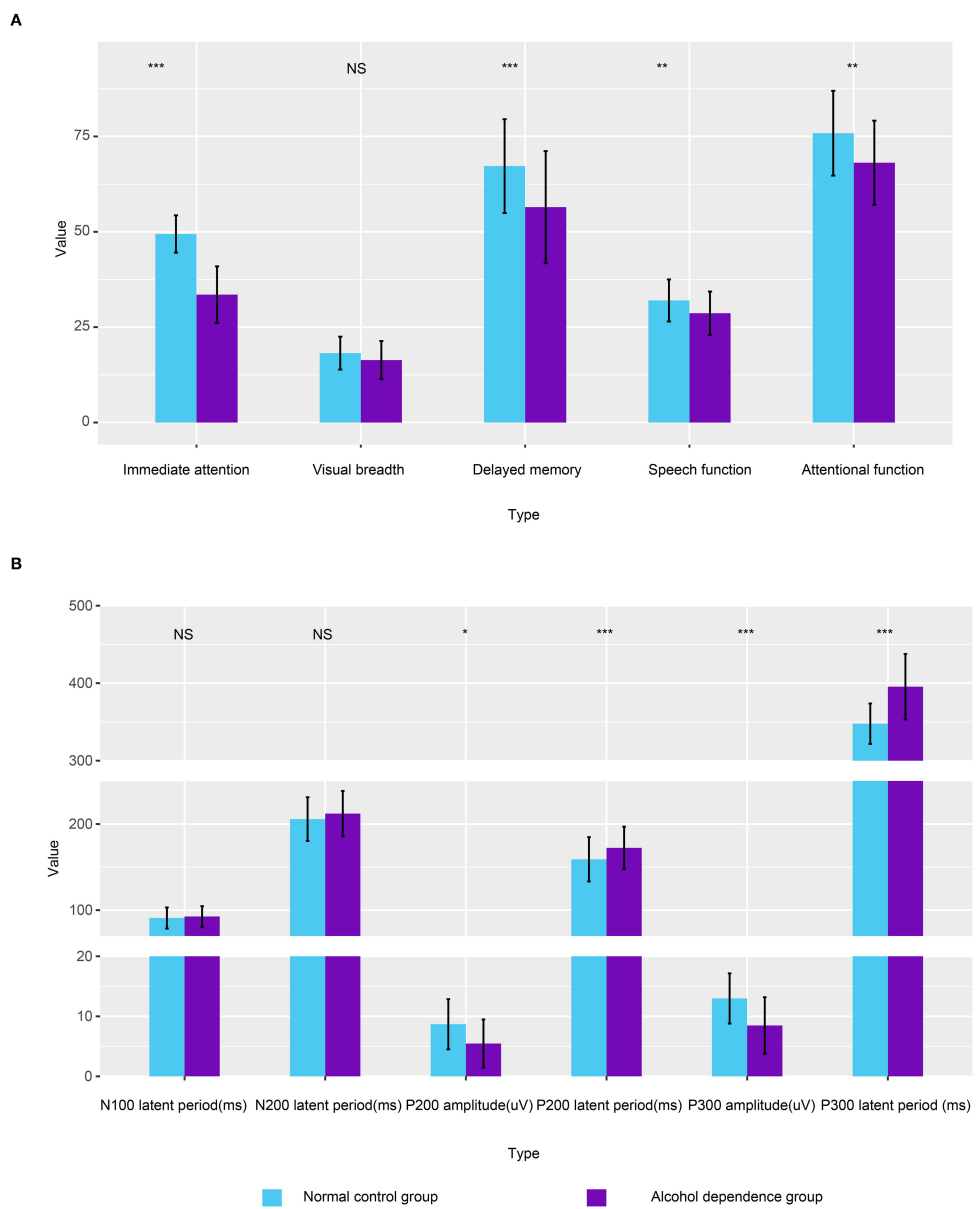
Column plot depicting the differences of alcohol-dependence group and the normal control group. **(A)** The correlation between the first-drinking age, alcohol dependence years, daily alcohol consumption, and RBANS scores in the alcohol-dependence group. **(B)** Comparison of ERPs between the alcohol-dependence group and the normal control group. Error bars refer to the SEM of each variable.

Bonferroni correction was conducted by the number of comparisons. *P* < 0.01 was considered statistically significant. Compared with the normal control, the original scores of speech function, attention function, delayed memory, and immediate attention in the alcohol-dependent group are significantly reduced (Figure 1A; *t* = −2.918, *t* = −3.426, *t* = −3.822, *t* = −12.928; *P* = 0.004, 0.001, 0.000, 0.000, respectively); Compared with the normal control, there is no significant difference in the original score of visual breadth in the alcohol-dependent group (*t* = −1.895, *P* = 0.054), as seen in Table 2.

**Table 2:** Comparison of the original score in RBANS (mean ± SD).

	**Alcohol-dependence group**	**Normal control group**	***t***	***p***
Visual breadth	16.37 ± 5.00	18.20 ± 4.31	−1.895	0.054
Speech function	28.65 ± 5.70^*^	32.00 ± 5.51	−2.918	0.004
Attentional function	68.12 ± 11.02^*^	75.85 ± 11.12	−3.426	0.001
Delayed memory	56.47 ± 14.69^*^	67.23 ± 12.30	−3.822	0.000
Immediate attention	33.53 ± 7.41^*^	49.43 ± 4.89	−12.928	0.000

* *p < 0.01*.

### The Correlation Between the First Drinking Age, Alcohol Dependence Years, Daily Alcohol Consumption, and RBANS Scores in the Alcohol Dependence Group

The correlation between the first-drinking age, the age at alcohol dependence, and the amount of daily alcohol consumption in the alcohol-dependence group and the RBANS scores are shown in Table 3. Notably, items from RBANS scores have no statistically significant correlation with the three alcohol-related factors.

**Table 3:** Analysis of the correlation between the first-drinking age, year of alcohol dependence, daily alcohol consumption, and RBANS scores in the alcohol-dependence group.

	**First-drinking age**	**Year of alcohol dependence**	**Daily alcohol consumption**
Visual breadth	−0.031	0.054	0.020
Speech function	0.079	−0.108	0.004
Attentional function	−0.019	0.189	0.119
Delayed memory	−0.145	−0.154	−0.056
Immediate attention	0.009	0.069	0.224

*p < 0.05.

### Comparison of ERPs Between the Alcohol Dependence Group and the Normal Control Group

Bonferroni correction was conducted by the number of comparisons. *P* < 0.008 was considered statistically significant. The results show that the incubation period of P300 in the alcohol-dependence group is significantly longer than that of the normal control group (Figure 1B; *t* = −6.986, *P* = 0.000). The differences in the incubation periods of N100 and N200 in the two groups are not statistically significant (*t* = −0.716, *t* = −1.208, *P* < 0.05), as seen in Table 4. The correlation between the first-drinking age, the age at alcohol dependence, and the amount of daily alcohol consumed in the alcohol-dependence group and the ERPs is shown in Table 5.

**Table 4:** Comparison of event-related potentials between the alcohol-dependence group and normal control groups (mean ± SD).

	**Alcohol-dependence group**	**Normal control groups**	***t***	***p***
N100 latent period (ms)	92.62 ± 11.98	90.85 ± 12.26	0.716	0.476
N200 latent period (ms)	212.12 ± 26.29	205.73 ± 25.34	1.208	0.230
P200 latent period (ms)	172.30 ± 24.64^*^	159.03 ± 25.73	2.593	0.010
P200 amplitude (μV)	5.47 ± 4.01^*^	8.68 ± 4.18	−3.853	0.000
P300 latent period (ms)	395.43 ± 42.25^*^	347.68 ± 26.06	6.986	0.000
P300 amplitude (μV)	8.47 ± 4.71^*^	12.97 ± 4.18	−5.012	0.000

* *p < 0.008*.

**Table 5:** Analysis of the correlation between the first-drinking age, year of alcohol dependence, daily alcohol consumption, and ERPs in the alcohol-dependence group.

	**First-drinking age**	**Year of alcohol dependence**	**Daily alcohol consumption**
N100 latent period (ms)	0.197	0.204	−0.181
N200 latent period (ms)	−0.051	−0.033	−0.004
P200 latent period (ms)	−0.159	−0.049	−0.050
P200 amplitude (μV)	−0.092	−0.051	0.066
P300 latent period (ms)	−0.108	−0.036	−0.075
P300 amplitude (μV)	−0.113	−0.117	0.031

*p < 0.05.

### Correlation Between RBANS Scores and ERPs in the Alcohol Dependence Group

**Figure 2:**
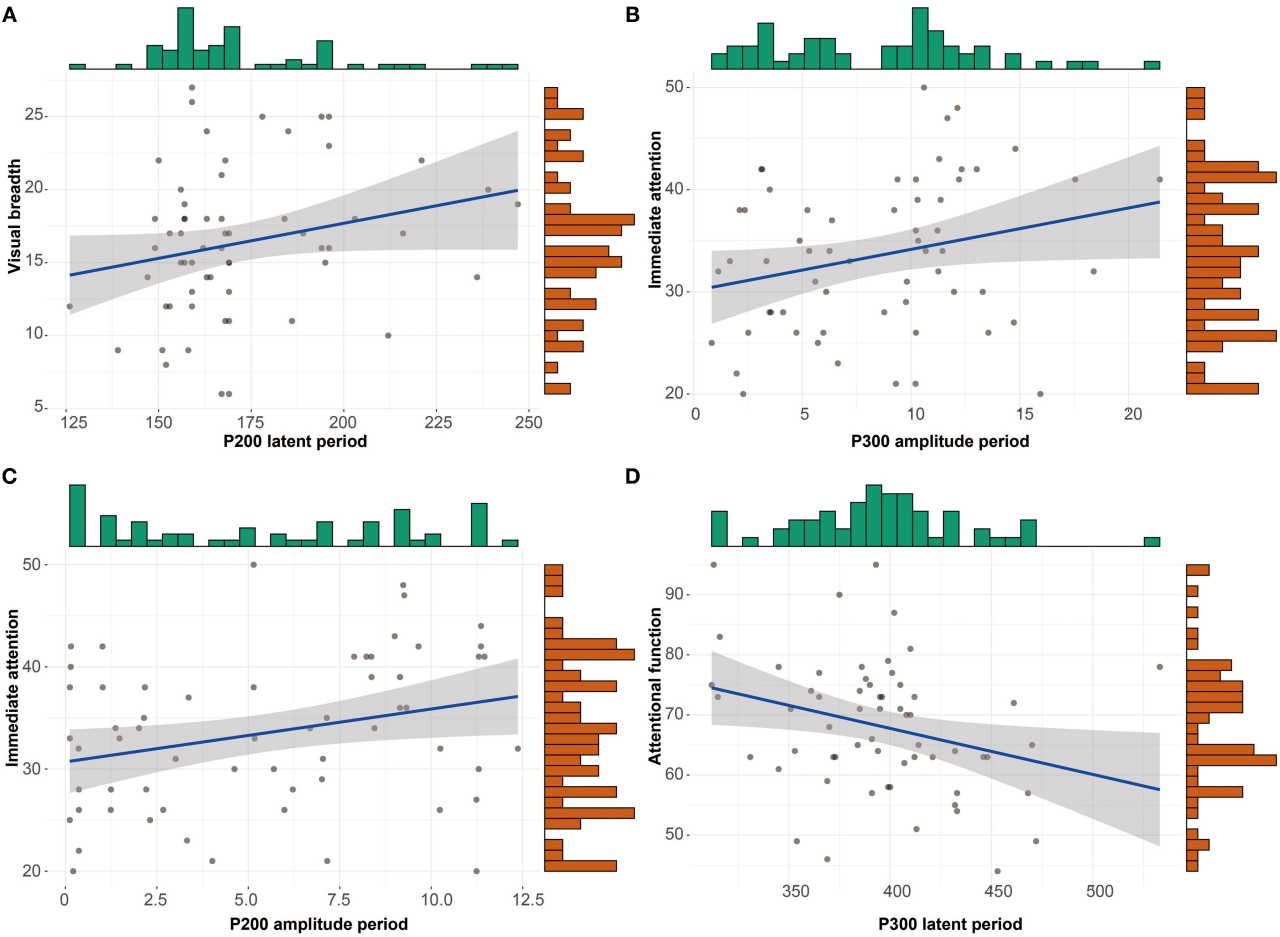
Scattering plot depicting the correlation between items in RBANS and items in ERPs with statistical significance. **(A)** Correlation between visual breadth and P200 latent period. **(B)** Correlation between immediate attention and P300 amplitude period. **(C)** Correlation between immediate attention and P200 amplitude period. **(D)** Correlation between attentional function and P300 latent period.

The correlations between RABNS scale scores and the components of ERPs were analyzed in the alcohol-dependence group by partial correlation analysis that has adjusted for confounding covariates, including age, education, age at first drinking, daily drinking, alcohol dependence years, and the daily amount of smoking, as seen in Tables 6, 7. The results show that the visual breadth is positively correlated with the P200 incubation period (Figure 2A, *r* = 0.278, *P* = 0.043). The immediate attention in the alcohol-dependence group is positively correlated with the amplitudes of P300 and P200 (Figures 2B,C; *r* = 0.282, *r* = 0.307; *P* = 0.041, 0.025). The attentional function is negatively correlated with the P300 latent period (Figure 2D, *r* = −0.338, *P* = 0.013).

**Table 6:** Correlation analysis between RBANS scores and ERPs in alcohol-dependence group (*r*).

	**P300 latent period**	**P300 amplitude**	**P200 latent period**	**P200 amplitude**	**N100 latent period**	**N200 latent period**
Immediate attention	−0.204	0.282^*^	−0.175	0.307^*^	−0.107	−0.151
Visual breadth	−0.006	−0.079	0.278^*^	−0.144	−0.248	0.248
Speech function	0.130	−0.139	0.046	−0.113	−0.004	0.110
Attentional function	−0.338^*^	0.077	−0.098	0.135	0.206	−0.103
Delayed memory	−0.001	0.004	0.217	0.002	−0.226	0.175

* *p < 0.05*.

**Table 7:** Correlation analysis between RBANS scores and ERPs in alcohol-dependence group (*p*).

	**P300 latent period**	**P300 amplitude**	**P200 latent period**	**P200 amplitude**	**N100 latent period**	**N200 latent period**
Immediate attention	0.143	0.041^*^	0.211	0.025^*^	0.444	0.280
Visual breadth	0.966	0.576	0.043^*^	0.304	0.073	0.073
Speech function	0.355	0.321	0.741	0.420	0.978	0.430
Attentional function	0.585	0.013^*^	0.337	0.484	0.464	0.139
Delayed memory	0.978	0.994	0.990	0.119	0.210	0.103

* *p < 0.05*.

## Discussion

In this study, we confirmed that the alcohol-dependence group had significant impairments in immediate attention, attentional function, delayed memory, and speech function. Further, an abnormality of P300 in alcohol-dependent people was observed, which was characterized by an extended incubation period and a decrease in amplitude. A significantly positive correlation was finally obtained between items in ERPs and RBANS scale, indicating the interconnection between these two assessment tools.

Previous studies have already shown that alcohol impaired an individual's attention function, creating a “short-sighted” effect, as a result of drinkers being able to pay only limited attention to some of the clues (9). In addition, under the influence of impaired attention ability, the activating and monitoring balance system of drinkers was disrupted, which further affected their cognitive and behavioral abilities (7, 24). Moreover, some scholars found that alcohol-dependent people could not completely reverse their impaired cognitive function even after temperance (25, 26). These studies supported that alcohol dependents may have multidisciplinary cognitive impairments. Our study provided further evidence that alcohol-dependent people had significant impairments in immediate attention, attention function, delayed memory, and speech function, indicating that the severity of cognitive impairment in alcohol dependents may be involved in language, attention function, and other cognitive fields.

Further, previous studies supported that the incubation period of P300 of patients with alcohol dependence was extended, and amplitude of P300 was reduced, compared to healthier people, indicating that their information-processing capacities were worse, and their cognitive functions were decreased compared with that of healthy subjects (27, 28), which was consistent with our findings. These findings suggest that auditory P300 could serve as a trait marker for alcohol dependents. Moreover, Park et al. showed that the P300 amplitude in the parietal area and the central region of the alcohol-dependence group was lower than that of the normal group (29). Restricted to the experimental design in this study, future research is expected to locate the different regions of subjects to improve the reliability and comprehensiveness of results.

The study also found no significant difference in the incubation period of N200 in the alcohol-dependent group compared to the normal group, which was consistent with Crego's report (30). However, there was an opposite view that the incubation period of N200 was prolonged in alcohol dependents. Maurage et al. compared ERPs in binge drinkers and normal subjects, showing no significant difference in baseline values for the latency periods of N200, P300, and P100 in binge drinkers, but a significant increase in the incubation period when measured again 9 months later. It was believed that the latency changes caused by severe AUD in a short period of time were similar to alcohol dependence, reflecting a pathological slowdown in information processing (31). In other words, the body intake of a large amount of alcohol in a short period of time may induce obvious brain dysfunction.

Although many previous studies on the cognitive function of alcohol dependence have similar and robust conclusions about P300 and N200, there were few reports on N100 and P200 in alcohol dependence. Considered an exogenous component, N100 occurs without effortful task demands. N100 is positioned in the primary auditory cortex and modulated by attention, as a reliable index to evaluate the subjects' selective attention and working memory (32). P200 may be produced in the primary and secondary auditory cortex, mainly reflecting the early stages of perceptual processing (33). Our results showed that there was no significant difference between the incubation period of N100 component between the alcohol-dependence group and the normal group, but a significant difference between the incubation period and the amplitude of P200. These results suggested that the P200-related sensory cortex of alcohol-dependent people may be impaired, leading to impaired perception and attention function in patients.

Our study, first, combined the RBANS scale with ERPs to evaluate cognitive impairment in alcohol-dependent Chinese. The correlation analysis showed that the incubation period of P300 in alcohol-dependent group was negatively correlated with attention function, and amplitudes of P300 and P200 were positively correlated with immediate attention score. Previous study has reported a decrease in visual N100 amplitudes in individuals with AUD (34). Consistently, our finding indicated that the incubation period of N100 was positively correlated with visual breadth. These results showed that with the increased amplitude of P300 and P200, the immediate attention function of alcohol dependents was aggravated. With the extended incubation period of P300 and P200, attention function and visual breadth were gradually alleviated and aggravated, respectively. Notably, Kim and Lee (35) observed that in 25 alcohol-dependent patients the incubation period of P300 was prolonged and the cognitive ability screening test score decreased, in line with our results (35). This suggested that the latency period of P200, P300, and amplitude of P300 had good consistency in regard to the scale scoring results, verifying each other on cognitive function tests.

Although our study was not yet certain whether ERPs were directly involved in the cognitive process, based on the correlation between ERPs and RBANS scores, ERPs with multicomponent had its unique advantages in comprehensively understanding the electrophysiological characteristics in cognitive impairment among alcohol-dependent people, providing a relatively objective criteria for the diagnosis of cognitive impairment in alcohol dependents. However, there are also several limitations to this study. One major limitation is that the study included only male participants, which limits the generalizability of the results. Another limitation is that this study is a cross-sectional study without longitudinal observation of alcohol dependents. Moreover, the alcohol-dependence group and normal control group are not strictly intelligence quotient (IQ) linked, and education year is insufficient to represent the IQ level of subjects that could potentially influence the accuracy of results. Recently, imaging is of great significance to the prevention, diagnosis, and treatment of cognitive impairment in alcohol dependents. But there are no imaging data used for the assessment of cognitive function in this study, and it needs to be further strengthened.

In summary, alcohol dependents have different degrees of cognitive impairment, which was characterized by immediate memory, attention function, delayed memory, and speech function. Using ERPs, alcohol-dependent cognitive impairment was characterized by an extended incubation period of P200, P300, and a decrease in amplitude. This study verified that ERPs and RBANS scales had good consistency in the evaluation of cognitive impairment, such that ERPs may be used as an objective and reliable neuroelectrophysiological index to assess cognitive impairment in alcohol-dependent people. Combined scale of RBANS and ERPs provided an objective basis for early diagnosis of alcohol dependence cognitive impairment.

## Data Availability Statement

The original contributions presented in the study are included in the article/supplementary materials, further inquiries can be directed to the corresponding author/s.

## Ethics Statement

This study was approved by the Ethics Committee of the Second People's Hospital of Hunan Province. The patients/participants provided their written informed consent to participate in this study.

## Author Contributions

XuZ and HC conceptualized and designed the research. ZL, WZ, MZ, and XL prepared the assessment tools. JX, JY, and XC performed the experiments. CH, SH, and XuZ undertook the statistical analysis. HC and XuZ wrote the first draft of the manuscript and contributed to the final manuscript. All authors critically reviewed the content, approved the final version for publication, and made substantial contributions to this study.

## Conflict of Interest

The authors declare that the research was conducted in the absence of any commercial or financial relationships that could be construed as a potential conflict of interest.

## References

[1] Staples MC Mandyam CD. Thinking after drinking: impaired hippocampal-dependent cognition in human alcoholics and animal models of alcohol dependence. Front Psychiatry. (2016) 7:162. 10.3389/fpsyt.2016.00162 27746746 PMC5043052

[2] Dhanabalan G Le Maitre TW Bogdanovic N Alkass K Druid H. Hippocampal granule cell loss in human chronic alcohol abusers. Neurobiol Dis. (2018) 120:63–75. 10.1016/j.nbd.2018.08.011 30189262

[3] Bernardin F Maheut-Bosser A Paille F. Cognitive impairments in alcohol-dependent subjects. Front Psychiatry. (2014) 5:78. 10.3389/fpsyt.2014.00078 25076914 PMC4099962

[4] Florez G Espandian A Villa R Saiz PA. Clinical implications of cognitive impairment and alcohol dependence. Adicciones. (2019) 31:3–7. 10.20882/adicciones.1284 30699230

[5] Mukherjee S Alcoholism and its effects on the central nervous system. Curr Neurovasc Res. (2013) 10:256–62. 10.2174/15672026113109990004 23713737

[6] Olaithe M Weinborn M Lowndes T Ng A Hodgson E Fine L. Repeatable battery for the assessment of neuropsychological status (RBANS): normative data for older adults. Arch Clin Neuropsychol. (2019) 34:1356–66. 10.1093/arclin/acy102 30608541

[7] Zhang XY Tan YL Chen DC Tan SP Yang FD Zunta-Soares GB. Effects of cigarette smoking and alcohol use on neurocognition and BDNF levels in a Chinese population. Psychopharmacology. (2016) 233:435–45. 10.1007/s00213-015-4124-6 26518023

[8] Brown P Heirene RM Gareth Roderique D John B Evans JJ. Applicability of the ACE-III and RBANS cognitive tests for the detection of alcohol-related brain damage. Front Psychol. (2019) 10:2636. 10.3389/fpsyg.2019.02636 31849759 PMC6892773

[9] Green A Garrick T Sheedy D Blake H Shores EA Harper C. The effect of moderate to heavy alcohol consumption on neuropsychological performance as measured by the repeatable battery for the assessment of neuropsychological status. Alcohol Clin Exp Res. (2010) 34:443–50. 10.1111/j.1530-0277.2009.01108.x 20028356

[10] Shura RD Brearly TW Rowland JA Martindale SL Miskey HM Duff K. RBANS validity indices: a systematic review and meta-analysis. Neuropsychol Rev. (2018) 28:269–84. 10.1007/s11065-018-9377-5 29770912

[11] Batty RA Francis A Thomas N Hopwood M Ponsford J Rossell SL. A brief neurocognitive assessment of patients with psychosis following traumatic brain injury (PFTBI): use of the repeatable battery for the assessment of neuropsychological status (RBANS). Psychiatry Res. (2016) 237:27–36. 10.1016/j.psychres.2016.01.062 26921048

[12] Duff K McDermott D Luong D Randolph C Boxer AL. Cognitive deficits in progressive supranuclear palsy on the repeatable battery for the assessment of neuropsychological status. J Clin Exp Neuropsychol. (2019) 41:469–75. 10.1080/13803395.2019.1572073 30712468 PMC6499681

[13] Campanella S. Why it is time to develop the use of cognitive event-related potentials in the treatment of psychiatric diseases. Neuropsychiatr Dis. Treat. (2013) 9:1835–45. 10.2147/NDT.S53687 24348040 PMC3849081

[14] Morrison C Rabipour S Knoefel F Sheppard C Taler V. Auditory event-related potentials in mild cognitive impairment and Alzheimer's disease. Curr Alzheimer Res. (2018) 15:702–15. 10.2174/1567205015666180123123209 29359668

[15] Campanella S Schroder E Kajosch H Noel X Kornreich C. Why cognitive event-related potentials (ERPs) should have a role in the management of alcohol disorders. Neurosci Biobehav Rev. (2019) 106:234–44. 10.1016/j.neubiorev.2018.06.016 29936112

[16] Maurage P Philippot P Verbanck P Noel X Kornreich C Hanak C. Is the P300 deficit in alcoholism associated with early visual impairments (P100, N170)? An oddball paradigm. Clin Neurophysiol. (2007) 118:633–44. 10.1016/j.clinph.2006.11.007 17208045

[17] Hamidovic A Wang Y. The P300 in alcohol use disorder: a meta-analysis and meta-regression. Prog Neuropsychopharmacol Biol Psychiatry. (2019) 95:109716. 10.1016/j.pnpbp.2019.109716 31369766

[18] Helfrich RF Knight RT. Cognitive neurophysiology: event-related potentials. Handb Clin Neurol. (2019) 160:543–58. 10.1016/B978-0-444-64032-1.00036-9 31277875

[19] Bagga D Khushu S Modi S Kaur P Bhattacharya D Garg ML. Impaired visual information processing in alcohol-dependent subjects: a proton magnetic resonance spectroscopy study of the primary visual cortex. J Stud Alcohol Drugs. (2014) 75:817–26. 10.15288/jsad.2014.75.817 25208200

[20] Dickter CL Forestell CA Hammett PJ Young CM. Relationship between alcohol dependence, escape drinking, and early neural attention to alcohol-related cues. Psychopharmacology. (2014) 231:2031–40. 10.1007/s00213-013-3348-6 24292342 PMC3988240

[21] Ram D George M Gowdappa B. Correlation of cognitive functions with emotional dysregulation in alcohol dependence: a preliminary study. Indian J Psychiatry. (2018) 60:307–11. 10.4103/psychiatry.IndianJPsychiatry_183_18 30405256 PMC6201679

[22] Pelletier S Alarcon R Ewert V Forest M Nalpas B Perney P. Comparison of the MoCA and BEARNI tests for detection of cognitive impairment in in-patients with alcohol use disorders. Drug Alcohol Depend. (2018) 187:249–53. 10.1016/j.drugalcdep.2018.02.026 29684893

[23] Randolph C Tierney MC Mohr E Chase TN. The repeatable battery for the assessment of neuropsychological status (RBANS): preliminary clinical validity. J Clin Exp Neuropsychol. (1998) 20:310–9. 10.1076/jcen.20.3.310.823 9845158

[24] Naim-Feil J Fitzgerald PB Bradshaw JL Lubman DI Sheppard D. Neurocognitive deficits, craving, and abstinence among alcohol-dependent individuals following detoxification. Arch Clin Neuropsychol. (2014) 29:26–37. 10.1093/arclin/act090 24334264

[25] Kaur P Sidana A Malhotra N Gupta A. Effects of abstinence of alcohol on neurocognitive functioning in patients with alcohol dependence syndrome. Asian J Psychiatr. (2020) 50:101997. 10.1016/j.ajp.2020.101997 32145693

[26] Nowakowska-Domagala K Jablkowska-Gorecka K Mokros L Koprowicz J Pietras T. Differences in the verbal fluency, working memory and executive functions in alcoholics: short-term vs. long-term abstainers. Psychiatry Res. (2017) 249:1–8. 10.1016/j.psychres.2016.12.034 28063392

[27] Chwedorowicz R Raszewski G Kapka-Skrzypczak L Sawicki K Studzinski T. Event-related potentials (ERP) and SGIP1 gene polymorphisms in alcoholics: relation to family history of alcoholism and drug usage. Ann Agric Environ Med. (2016) 23:618–24. 10.5604/12321966.1226856 28030933

[28] Kreusch F Quertemont E Vilenne A Hansenne M. Alcohol abuse and ERP components in Go/No-go tasks using alcohol-related stimuli: impact of alcohol avoidance. Int J Psychophysiol. (2014) 94:92–9. 10.1016/j.ijpsycho.2014.08.001 25110836

[29] Park M Kim YJ Kim DJ Choi JS. Differential neurophysiological correlates of information processing in Internet gaming disorder and alcohol use disorder measured by event-related potentials. Sci Rep. (2017) 7:9062. 10.1038/s41598-017-09679-z 28831146 PMC5567258

[30] Crego A Cadaveira F Parada M Corral M Caamano-Isorna F Rodriguez Holguin S. Increased amplitude of P3 event-related potential in young binge drinkers. Alcohol. (2012) 46:415–25. 10.1016/j.alcohol.2011.10.002 22459872

[31] Maurage P Pesenti M Philippot P Joassin F Campanella S. Latent deleterious effects of binge drinking over a short period of time revealed only by electrophysiological measures. J Psychiatry Neurosci. (2009) 34:111–8. 10.1016/S1053-8119(09)70040-0 19270761 PMC2647570

[32] Cohen HL Ji J Chorlian DB Begleiter H Porjesz B. Alcohol-related ERP changes recorded from different modalities: a topographic analysis. Alcohol Clin Exp Res. (2002) 26:303–17. 10.1111/j.1530-0277.2002.tb02539.x 11923582

[33] Lijffijt M Cox B Acas MD Lane SD Moeller FG Swann AC. Differential relationships of impulsivity or antisocial symptoms on P50, N100, or P200 auditory sensory gating in controls and antisocial personality disorder. J Psychiatr Res. (2012) 46:743–50. 10.1016/j.jpsychires.2012.03.001 22464943 PMC3667738

[34] Patterson BW Williams HL McLean GA Smith LT Schaeffer KW. Alcoholism and family history of alcoholism: effects on visual and auditory event-related potentials. Alcohol. (1987) 4:265–74. 10.1016/0741-8329(87)90022-X 3620095

[35] Kim SH Lee YH. Correlation between cognitive capacity screening examination and cognitive evoked potential in alcohol-dependent patients. Yonsei Med J. (2004) 45:796–802. 10.3349/ymj.2004.45.5.796 15515188

